# MCR-1 Confers Cross-Resistance to Bacitracin, a Widely Used In-Feed Antibiotic

**DOI:** 10.1128/mSphere.00411-18

**Published:** 2018-09-05

**Authors:** Fuzhou Xu, Ximin Zeng, Atsushi Hinenoya, Jun Lin

**Affiliations:** a Department of Animal Science, The University of Tennessee, Knoxville, Tennessee, USA; b Institute of Animal Science and Veterinary Medicine, Beijing Academy of Agriculture and Forestry Sciences, Beijing, China; c Graduate School of Life and Environmental Sciences, Osaka Prefecture University, Japan; Escola Paulista de Medicina/Universidade Federal de São Paulo

**Keywords:** bacitracin, colistin resistance, cross-resistance, feed additive, risk factor

## Abstract

Extensive use of colistin in food animals is deemed a major driving force for the emergence and transmission of *mcr-1*. However, a non-colistin usage factor(s) contributing to mobile colistin resistance may also exist in animal production systems. Given that polymyxin, a bacterium-derived peptide antibiotic, has been successfully used as a surrogate to study bacterial resistance to antimicrobial peptides (AMPs), acquisition of MCR-1 may confer cross-resistance to the unrelated AMPs implicated in practical applications. To test this, we first constructed Escherichia coli recombinant strains differing only in the presence or absence of functional MCR-1. Among diverse tested AMPs, MCR-1 was observed to confer cross-resistance to bacitracin, an in-feed antibiotic widely used in animal industry. The significantly (2-fold) increased bacitracin MIC was confirmed by using different bacitracin products, broth media, and laboratory host strains for susceptibility tests. Subsequently, an original *mcr-1* gene-bearing plasmid, pSLy21, was conjugatively transferred to eight clinical E. coli recipient strains isolated from diarrheic pigs, which also led to significantly increased MICs of both colistin (4-fold to 8-fold) and bacitracin (2-fold). Growth curve examination further demonstrated that MCR-1 provides a growth advantage to various E. coli strains in the presence of bacitracin. Given that bacitracin, a feed additive displaying low absorption in the intestine, can be used in food animals with no withdrawal required, imprudent use of bacitracin in food animals may serve as a risk factor to enhance the ecological fitness of MCR-1-positive E. coli strains, consequently facilitating the persistence and transmission of plasmid-mediated colistin resistance in agricultural ecosystem.

**IMPORTANCE** Polymyxins (e.g., colistin) are the drugs of last resort to treat multidrug-resistant infections in humans. To control mobile colistin resistance, there is a worldwide trend to limit colistin use in animal production. However, simply limiting colistin use in animal production may still not effectively mitigate colistin resistance due to an overlooked non-colistin usage factor(s). Using controlled systems, in this study, we observed that MCR-1 confers cross-resistance to bacitracin, a popular in-feed antibiotic used in food animals. Thus, imprudent and extensive usage of bacitracin in food animals may serve as a non-colistin usage risk factor for the transmissible colistin resistance. Further comprehensive *in vitro* and *in vivo* studies are highly warranted to generate science-based information for risk assessment and risk management of colistin resistance, consequently facilitating the development of proactive and effective strategies to mitigate colistin resistance in animal production system and protect public health.

## Abstract (precis)

Polymyxins (e.g., colistin) are the drugs of last resort to treat multidrug-resistant infections in humans. To control mobile colistin resistance, there is a worldwide trend to limit colistin use in animal production. However, simply limiting colistin use in animal production may still not effectively mitigate colistin resistance due to an overlooked non-colistin usage factor(s). Using controlled systems, in this study, we observed that MCR-1 confers cross-resistance to bacitracin, a popular in-feed antibiotic used in food animals. Thus, imprudent and extensive usage of bacitracin in food animals may serve as a non-colistin usage risk factor for the transmissible colistin resistance. Further comprehensive *in vitro* and *in vivo* studies are highly warranted to generate science-based information for risk assessment and risk management of colistin resistance, consequently facilitating the development of proactive and effective strategies to mitigate colistin resistance in animal production system and protect public health.

## OBSERVATION

Polymyxins (e.g., colistin; also known as “polymyxin E”) are the drugs of last resort to treat multidrug-resistant infections in humans. Recent discovery of a novel mobile colistin resistance gene, *mcr-1*, has drawn worldwide attention and fear (1). Extensive usage of colistin in food animals is deemed a major driving force for the emergence and transmission of the *mcr-1* gene (1, 2, 3). Although limiting colistin usage in animal production (2, 3) is likely the most straightforward approach to mitigate transmissible colistin resistance, a non-colistin usage factor(s) contributing to the persistence and transmission of *mcr-1* gene may also exist in complex ecosystems (4). In the United States, despite lack of colistin usage in food animals, *mcr-1*-positive Escherichia coli strains were still isolated from swine intestinal samples (5).

As a bacterium-derived antimicrobial peptide (AMP), polymyxin has been widely used as an AMP surrogate to study mechanisms of bacterial resistance to host defense AMPs although polymyxin bears little structural resemblance to many AMPs (6–10). Acquisition of polymyxin resistance might result in cross-resistance to certain unrelated AMPs (6–10). This evidence prompted us to examine if acquisition of the polymyxin resistance determinant MCR-1 can confer increased resistance to other AMPs. In fact, Napier et al. (11, 12) revealed a positive correlation between resistance to colistin and resistance to the host AMP LL-37 and lysozyme. Later, Sherman et al. (13) demonstrated that colistin confers cross-resistance to lysozyme. In an independent study (14), MCR-1 was not observed to confer cross-resistance to three human AMPs; however, due to the diverse backgrounds of the tested strains in this study (14), the findings were likely obscured by confounding factors resulting from various levels of intrinsic AMP resistance of different strains. Thus, to definitively examine if MCR-1 confers cross-resistance to AMPs, well-controlled genetic systems are critically needed and were used in this study.

We first constructed two Escherichia coli recombinant strains with the same genetic background that had differences solely in MCR-1 expression levels. Briefly, the *mcr-1* gene was PCR amplified from a *mcr-1*-positive swine E. coli strain (GenBank sequence accession no. CP015912) (5) using primers mcr-1_F (ATGATGCAGCATACTTCTGTGTG) and mcr-1_R (CGCGGATCCTCAGCGGATGAATGCG). PCR was performed using *Pfu*Ultra DNA polymerase (Stratagene). The blunt-ended PCR product was digested with BamHI and cloned into expression vector pZE21 (15) digested with both BamHI and EcoRV, creating recombinant plasmid pMCR-1. The pZE21 and pMCR-1 plasmids were then individually transformed into E. coli Top10 strains. The MICs of colistin (Table 1) for constructs Top10/pMCR-1 and Top10/pZE21 were 8 µg/ml and 1 µg/ml, respectively; the MICs were determined using the broth microdilution method recommended by the CLSI (16).

**TABLE 1:** Colistin and bacitracin MICs for various *E. coli* strains and constructs

Strain	MIC		Source
	Colistin (µg/ml)^*a*^	Bacitracin (mg/ml)^*b*^	
Laboratory strains			
TOP10	1	1	Invitrogen (catalog no. C4040-03)
TOP10/pZE21	1	1	This study
TOP10/pMCR-1	8	2	This study
DH5α	1	1	Invitrogen (catalog no. 18263012)
DH5α/pZE21	1	1	This study
DH5α/pMCR-1	8	2	This study
Clinical strains			
3030-2	1	1	18
3030-2/pSLy21	8	2	This study
8508	1	1	17
8508/pSLy21	4	2	This study
8510	1	2	17
8510/pSLy21	8	4	This study
8511	1	1	17
8511/pSLy21	8	2	This study
8512	1	1	17
8512/pSLy21	8	2	This study
8518	1	2	17
8518/pSLy21	8	4	This study
8532	1	1	17
8532/pSLy21	8	2	This study
8537	1	2	17
8537/pSLy21	8	4	This study
Str^r^MG1655	1	2	Tyrrell Conway
Str^r^MG1655/pSLy21	8	4	This study

a The colistin sulfate salt was purchased from Acros Organics (catalog no. 15565146).

b The bacitracin, which has high solubility in water (50 mg/ml), was purchased from Sigma-Aldrich (catalog no. 11702).

**FIG 1:**
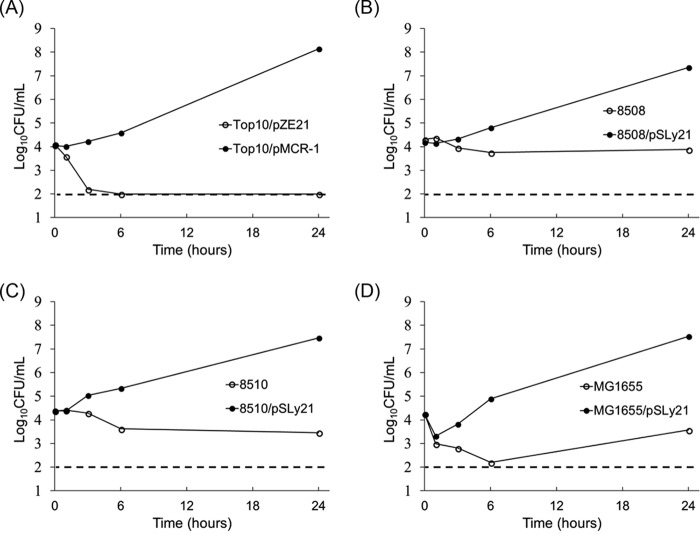
Effect of acquisition of MCR-1 on the growth of E. coli in the presence of bacitracin. The *in vitro* growth of laboratory E. coli Top10/pZE21and Top10/pMCR-1 isolates (panel A) and clinical E. coli isolates together with their corresponding derivatives carrying pSLy21 plasmid (panels B to D) was examined in Luria-Bertani (LB) broth supplemented with bacitracin. Similar amounts of E. coli cells (late log phase) were inoculated in LB broth supplemented with bacitracin (in panels A and B, 1 mg/ml; in panels C and D, 2 mg/ml) and grown at 37°C. The detection limit of the method was 100 CFU/ml (dashed line). Each data point represents the mean value obtained from triplicate wells in the microtiter plate growth assay.

Subsequently, the susceptibilities of these two recombinant strains to a panel of diverse AMPs were examined using the same broth microdilution method. Most of the tested AMPs (corresponding producers), which included bacitracin (Bacillus licheniformis), gramicidin (soil bacterium), magainin (frog), protamine (salmon), and cecropin (moth), were purchased from Sigma. The chicken cathelicidin fowlicidin-1 was synthesized by Bio-Synthesis. Compared to the control Top10/pZE21 strain, the Top10/pMCR-1 strain did not show increased resistance to most tested AMPs, which included fowlicidin-1 (MIC = 16 µg/ml), protamine (MIC = 128 µg/ml), cecropin A (MIC = 16 µg/ml), and magainin and gramicidin (both with MICs of >32 µg/ml due to a solubility issue). However, the Top10/pMCR-1 strain showed a significantly (2-fold) increased bacitracin MIC (Table 1); exactly the same magnitude of increase in the bacitracin MIC was further confirmed by using different broth media for MIC tests (Muller-Hinton broth and Luria-Bertani broth); by using another bacitracin product (Sigma; catalog no. B5150) that displays low-level water solubility; and by using a different host strain, DH5α (Table 1). Notably, the bacitracin MIC increase is not attributable to intertest variability because all relevant strains were tested in duplicate within same microtiter plate for each independent MIC test; more importantly, the 2-fold MIC increase was also confirmed in at least three independent MIC tests. We also examined *in vitro* growth curves, which showed that the growth of the control Top10/pZE21 strain was greatly inhibited in the presence of bacitracin; after 6 h of incubation, no viable Top10/pZE21 cells could be detected (Fig. 1A). In contrast, the Top10/pMCR-1 strain grew normally in the presence of bacitracin at the same concentration.

Although the findings described above, obtained by using controlled genetic manipulation in laboratory E. coli strains, provided compelling evidence that MCR-1 confers cross-resistance to bacitracin, it was still important to determine if the original *mcr-1* gene-bearing plasmid can also confer increased bacitracin resistance in clinical E. coli strains. To test this, pSLy21, a 63-kb plasmid bearing *mcr-1* in a colistin-resistant U.S. swine isolate (5), was conjugatively transferred to eight porcine E. coli strains (17, 18) as well as to an E. coli MG1655 streptomycin-resistant (Str^r^) derivative; all selected and desired transconjugants were confirmed by pulsed-field gel electrophoresis (PFGE) analysis (data not shown). As shown in Table 1, acquisition of pSLy21 also led to a significantly (2-fold) increased bacitracin MIC in all clinical E. coli strains; this increase has also been confirmed in at least three independent MIC tests. In addition, the growth curve in the presence of bacitracin of three randomly selected E. coli clinical strains carrying pSLy21 clearly showed that pSLy21 conferred a growth advantage to the E. coli strains in the presence of bacitracin (Fig. 1B to D).

E. coli strains generally have high intrinsic resistance to bacitracin. Thus, to prevent and control bacterial infections in food animals, bacitracin is used primarily by targeting Gram-positive organisms rather than Gram-negative bacteria, such as E. coli. However, it is important that bacitracin can be used as an in-feed antibiotic over a long period at a high level in food animals (primarily swine and poultry). For example, the popular in-feed bacitracin product BMD (bacitracin methylene disalicylate; Zoetis) is recommended for use with no withdrawal required, regardless of whether the intended use is growth promotion (10 to 30 ppm in complete feed) or disease control (250 ppm) (19). In addition, bacitracin is absorbed only minimally in the gastrointestinal tract, with about 95% accumulating in the intestine (20), which may lead to a high concentration of bacitracin in specific niches in the intestine (e.g., at levels corresponding to milligrams per milliliter in cecum) and even in the environment due to long-term use of high levels of bacitracin. Thus, the potential risk of transmissible colistin resistance as a consequence of bacitracin usage in animal production is not an artificial scenario and needs to be assessed comprehensively by using well-controlled *in vitro* and *in vivo* systems in the future.

Since the discovery of MCR-1 in 2016 (1), at least five MCR-1 homologues have been identified (4). In this study, we focused only on MCR-1 because MCR-1 is still the predominant determinant of transmissible colistin resistance (4). At present, there is a worldwide trend to limit colistin usage in animal husbandry to protect public health. However, simply limiting or banning the use of colistin in animal production may not fully solve this serious and challenging antibiotic resistance issue; several potential non-colistin usage risk factors for colistin resistance have been identified and were discussed in a recent review (4). The findings from this study suggest that imprudent and extensive usage of bacitracin in food animals is a non-colistin usage risk factor for transmissible colistin resistance. We believe that bacitracin will continue to contribute to animal health and human health in the future; however, given the potential risk of the use of bacitracin observed in this study, we may have to revisit current recommendations for bacitracin usage in animal production and develop proactive plans to minimize the risk of bacitracin usage with respect to promoting colistin resistance in the United States and worldwide.
